# Transcriptome Dynamics and Potential Roles of *Sox6* in the Postnatal Heart

**DOI:** 10.1371/journal.pone.0166574

**Published:** 2016-11-10

**Authors:** Chung-Il An, Yasunori Ichihashi, Jie Peng, Neelima R. Sinha, Nobuko Hagiwara

**Affiliations:** 1 Division of Cardiovascular Medicine, Department of Internal Medicine, University of California Davis, Davis, California, United States of America; 2 Department of Plant Biology, University of California Davis, Davis, California, United States of America; 3 Department of Statistics, University of California Davis, Davis, California, United States of America; Cincinnati Children's Hospital Medical Center, UNITED STATES

## Abstract

The postnatal heart undergoes highly coordinated developmental processes culminating in the complex physiologic properties of the adult heart. The molecular mechanisms of postnatal heart development remain largely unexplored despite their important clinical implications. To gain an integrated view of the dynamic changes in gene expression during postnatal heart development at the organ level, time-series transcriptome analyses of the postnatal hearts of neonatal through adult mice (P1, P7, P14, P30, and P60) were performed using a newly developed bioinformatics pipeline. We identified functional gene clusters by principal component analysis with self-organizing map clustering which revealed organized, discrete gene expression patterns corresponding to biological functions associated with the neonatal, juvenile and adult stages of postnatal heart development. Using weighted gene co-expression network analysis with bootstrap inference for each of these functional gene clusters, highly robust hub genes were identified which likely play key roles in regulating expression of co-expressed, functionally linked genes. Additionally, motivated by the role of the transcription factor Sox6 in the functional maturation of skeletal muscle, the role of Sox6 in the postnatal maturation of cardiac muscle was investigated. Differentially expressed transcriptome analyses between Sox6 knockout (KO) and control hearts uncovered significant upregulation of genes involved in cell proliferation at postnatal day 7 (P7) in the Sox6 KO heart. This result was validated by detecting mitotically active cells in the P7 Sox6 KO heart. The current report provides a framework for the complex molecular processes of postnatal heart development, thus enabling systematic dissection of the developmental regression observed in the stressed and failing adult heart.

## Introduction

Our understanding of mammalian embryonic/fetal heart development has significantly advanced in recent years [1–3]. By contrast, postnatal development and functional maturation of the heart has remained less investigated. Except for an increase in size and heart rate [4], changes in the postnatal heart appear less dramatic compared to the significant morphological transformations of the embryonic heart [1]. However, at the physiological and cellular levels, the heart undergoes significant changes after birth, including a shift in the mode of cardiomyocyte growth from hyperplasia to hypertrophy with multinucleation and polyploidization [5–7], a shift in energy source and accompanying changes in metabolic enzymes (from glycolysis to fatty acid oxidation) [8], isoform switching by alternative splicing (e.g. *Mef2c*) [9, 10], and an increase in coronary vasculature and capillary growth [11]. These observations clearly demonstrate that the seemingly static postnatal heart is actually undergoing coordinated and systematic changes to gain functional maturation. Importantly, the failing adult heart exhibits derailment from the coordinated functional adaptation attained by the postnatal heart by re-expressing fetal isoforms of contractile proteins in diseased adult heart [12, 13] and shifting back to glycolysis from fatty acid oxidation [8]. Therefore, uncovering how fetal type genes are suppressed in the early postnatal heart should provide valuable information on the molecular mechanisms that characterize failing adult hearts.

To begin to investigate how fetal genes are suppressed, we have analyzed the role of Sox6 in postnatal heart development. Sox6 is a member of the evolutionarily conserved Sox (SRY-box) transcription factor family functioning as a transcriptional activator or repressor in various cell types [14–17]. We have previously reported that the systemic loss of a functional Sox6 protein in mice resulted in progressive atrioventricular heart block and ultrastructural changes in both cardiac and skeletal muscle, leading to death within two weeks after birth [18]. These observations suggest that Sox6 plays an important role in functional maturation and maintenance of both cardiac and skeletal muscle. In the skeletal muscle of mice and zebra fish, Sox6 has been shown to play a critical role in fiber type specification of skeletal muscle as a transcriptional suppressor of slow fiber-specific genes [19–22]. Previously, we have generated Sox6^loxp/loxp^; MCK-Cre mice in which Sox6 is knocked out specifically in both skeletal muscle and heart. These mice showed a significant increase in slow fibers in skeletal muscle, but were viable [19] in contrast to the Sox6 null mice which showed an early postnatal lethal phenotype [18]. Recently, it has been demonstrated that Sox6 regulates cardiomyocyte proliferation and differentiation during cardiac development [23–25]. Taken together, these observations suggest that although Sox6 expression in muscle is not essential for survival, Sox6 regulates functional maturation of muscle, fiber type differentiation of skeletal muscle and possibly postnatal differentiation of cardiac muscle.

Previously, reports of transcriptomic analyses in postnatal heart as well as injury-induced cardiomyocyte regeneration highlighted complexity of gene expression and identified potential regulators of cardiomyocyte cell cycle [26–29]. However, detailed gene expression profiles within the network context at the organ level have not been fully investigated. In order to gain an integrated view of gene expression dynamics during postnatal heart development and also to investigate roles of Sox6 in the functional maturation of cardiac muscle, we performed time-series transcriptome analysis of control and Sox6 KO cardiac ventricle of neonatal through adult mice using a new bioinformatics pipeline which we established in this report. We first analyzed transcriptome data from control mice to identify functional clusters by principal component analysis (PCA) with self-organizing map (SOM) clustering [30]. We next used weighted gene co-expression network analysis [31] with bootstrap inference [32] for each functional cluster to identify highly robust hub genes as potential key regulatory genes. Finally, we compared transcriptome data between Sox6 KO and control mice to infer biological functions of Sox6 during postnatal heart development.

## Materials and Methods

### Ethics Statement

This study was carried out in strict accordance with the recommendations in the Guide for the Care and Use of Laboratory Animals of the National Institutes of Health. All animal work and care were performed under the guidelines of the Institutional Animal Care and Use Committee (IACUC) at the University of California, Davis (UC Davis). Specific approval for the mouse experiments was obtained with the protocol # 18055 “Mouse Models for Human Heart and Skeletal Muscle Disease”. All procedures were terminal, and all efforts were made to minimize suffering. Specifically, mice were sacrificed by cervical dislocation to minimize the suffering. Also, anesthesia was not used to eliminate a possible impact on gene expression when mice are being stressed.

### Animals

For transcriptome analyses, MCK-Cre (the Jackson Laboratory, Bar Harbor, ME, USA) and Sox6^loxp/loxp^; MCK-Cre male mice [19] were used as control and experimental, respectively. MCK-Cre mice were chosen to inactivate the *Sox6* gene in postnatal heart because MCK expression is known to increase to 40% of maximum levels at birth, reach maximum levels at postnatal day 10, and remain at a high level thereafter in rats [33], therefore, suitable for the gene inactivation targeted at early postnatal stages in rodents. Also, MCK-Cre mice have been frequently used for studying gene functions in the heart [34–39]. For validation experiments using immunohistochemistry, age-matched C57BL/6 mice were also used. Sox6^loxp/loxp^ mice were a kind gift from Dr. Veronique Lefebvre at the Cleveland Clinic [40].

### RNA isolation

Cardiac ventricles were collected from postnatal day 1 (P1), day 7 (P7), day 14 (P14), 1 month (P30), and 2 months (P60) mice, rinsed in phosphate buffered saline (PBS), flash frozen in liquid nitrogen and stored at -80°C until use. Three biological replicates were prepared for each time point. Total RNA was extracted using TRIzol reagent (Invitrogen, Carlsbad, CA, USA) following the manufacture’s instruction and then further purified using RNA Clean & Concentrator-5 (Zymo Research, Irvine, CA, USA). The integrity of RNA was confirmed using Bioanalyzer 2100 (Agilent Technologies, Santa Clara, CA, USA). For reverse transcription-quantitative PCR (RT-qPCR), total RNA was purified using Direct-zol RNA MiniPrep (Zymo Research) with DNase I treatment steps according to the manufacturer’s instructions.

### Microarray experiments

Preparation of cRNA probes, hybridization, scanning and data collection were performed at the Expression Analysis Core Facility at UC Davis following the protocols provided by Illumina, Inc. Labeled cRNA probes were hybridized with MouseWG-6 v2.0 Expression BeadChip (Illumina, Inc., San Diego, CA, USA). Hybridization was done in triplicates for each biological replicate of control and Sox6 KO samples. GCT files, which contain raw signal intensity data, were created from Illumina IDAT zip files using IlluminaExpressionFileCreator at GenePattern (http://www.broadinstitute.org/cancer/software/genepattern/) [41]. Microarray data reported in this article are available at Gene Expression Omnibus (http://www.ncbi.nlm.nih.gov/geo/query/acc.cgi?token=uxadaeqwzpojdkx&acc=GSE60958).

### Preprocessing of raw microarray data

Hybridization signals below background levels in each array were removed using a mean+2σ of negative control values calculated using negative control probes on the beadchip arrays as an intensity cutoff. Next, “Bad” and “No match” probes which do not match with the most recent gene annotation database were removed using the information from illuminaMousev2PROBEQUALITY in the illuminaMousev2.db [42]. Probe annotations were then updated using the information from illuminaMousev2SYMBOLREANNOTATED in the illuminaMousev2.db and Mouse Genome Informatics (MGI). For time course data analysis (clustering and gene co-expression network analysis: see below), data from all arrays were combined and the probes missing at one or more time points were eliminated. When one gene was represented by multiple probes, a probe with the highest intensity at P1 was selected for the following analyses. Quantile normalization was then applied using the normalizeBetweenArrays function in the limma package [43]. Time course analysis was conducted using 6784 probes in total, each probe representing a single gene. Normalization of the combined data was confirmed by a box plot (S1A Fig). To see the reproducibility and similarity between each sample, a multidimensional scaling (MDS) plot was created using the R stats package, cmdscale (S1B Fig).

### PCA with SOM clustering

To minimize influences from potential outliers in the three biological replicates, median intensity values were used. To focus on developmentally regulated genes whose expression was dynamically changed during postnatal heart development, only genes within top 30% coefficient of variation (CV) for expression across samples (i.e. highly differentially expressed genes across samples) were used for the subsequent analyses. Scaled expression values were used for a multilevel 3X6 hexagonal SOM [44, 45] to cluster these genes. 100 training iterations were used during clustering, over which the alpha learning rate decreased from 0.009 to 0.006. The final assignment of genes to winning units formed the basis of the gene clusters. The outcome of SOM clustering was visualized in PCA space where PC values were calculated based on the gene expression across samples using the R stats package, prcomp. As a reference, we also performed PCA and SOM clustering using mean intensity values which resulted in comparable clusters with the ones with mean intensity values (S2 Fig).

### Gene ontology (GO) term enrichment analysis

GO term enrichment was analyzed at AmiGO (http://amigo1.geneontology.org/cgi-bin/amigo/term_enrichment) [46] using MGI as a database filter and a 0.01 maximum p-value cutoff.

### Gene co-expression network analysis

Gene co-expression network analysis was performed using the weighted correlation network analysis (WGCNA) package [31] with a gene set identified in each SOM cluster. The soft thresholding power was chosen based on the criterion of approximate scale-free topology (fit index = 0.8, or 0.85 where applicable). Next, adjacency matrix with the selected soft thresholding power was calculated. To minimize effects of noise and spurious associations, the adjacency matrix was transformed into topological overlap matrix (TOM), and then TOM-based connectivity was calculated. For genes in each SOM cluster, we constructed 100 WGCNA networks based on 100 bootstrapped replications of the data, and ranked nodes according to TOM-based connectivity. We then searched for robust hub genes by calculating average ranking of nodes, and defined genes ranked in top 5% as hub genes (S3 Fig). We visualized a part of network using the igraph R package ver. 0.7.0.

### Identification of differentially expressed genes between Sox6 KO and control cardiac ventricles

Differential gene expression analysis between control and Sox6 KO mice was performed using the limma package [47]. Results were filtered using a 0.01 maximum false discovery rate (FDR) and a 1.5 minimum fold change.

### Enrichment analysis of the Sox6 binding motif

Promoter sequences (1 kb upstream sequences from transcription start site) of differentially expressed genes in Sox6 KO mice were obtained at Ensembl BioMart (http://asia.ensembl.org/biomart/martview), and repeat sequences were masked using RepeatMasker [48]. Enrichment of the Sox6 binding motif (JASPAR CORE MA0515.1) in the repeat-masked promoter sequences was analyzed using AME [49].

### RT-qPCR

cDNA was prepared using High Capacity cDNA Reverse Transcription Kit (Applied Biosystems, Foster City, CA, USA). Quantitative PCR (qPCR) was performed on an ABI Prism 7900HT Sequence Detection System (Applied Biosystems) using PrimeTime qPCR Assays (Integrated DNA Technologies, Coralville, IA, USA) and SensiFast Probe Hi-ROX Kit (Bioline, Taunton, MA, USA). *Rpl37a* and *Ubb* were used as reference genes for normalizing expression levels of tested genes. The two reference genes were chosen by analysis using NormFinder [50] which demonstrated that the combination of these two genes represented the most stably expressed genes between P1 and P60 ventricles among 7 genes tested (*Actb*, *Gapdh*, *Huwe1*, *Rpl37a*, *Rpl41*, *Hdac3*, and *Ubb*: data not shown). Raw threshold cycle (C_T_) values of these two genes were averaged according to the instructions of NormFinder, and the mean C_T_ values were used for further calculations. All statistical analyses were performed using the two-tailed Student's t-test. Information on PrimeTime qPCR Assays used is provided in S1 Table.

### Immunohistochemistry

Cardiac ventricles were collected from two wild type (C57BL/6) and three Sox6 KO mice at P7 and embedded in Tissue Freezing Medium (Triangle Biomedical Sciences, Durham, NC, USA). Cryosections (16 μm) were fixed in 4% paraformaldehyde and were incubated with phospho-histone H3 (Ser10) rabbit polyclonal antibody (#9701, Cell Signaling Technology, Danvers, MA, USA) at 400 fold dilution. As a secondary antibody, Alexa Fluor 555-conjugated goat anti-rabbit IgG (A-21429, Invitrogen) was used at 600 fold dilution. Counterstaining with 4’,6-diamidino-2-phenylindole (DAPI) was performed using VECTASHIELD mounting medium with DAPI (Vector Laboratories, Inc., Burlingame, CA, USA). Fluorescence images were obtained at the confocal microscope facility at the Genome and Biomedical Sciences Facility at UC Davis. Background staining was obtained using only secondary antibody (negative control), and no specific signal was observed from negative control sections of both wild type and Sox6 KO myocardium.

## Results

### Dynamic gene expression patterns during postnatal heart development

**Fig 1:**
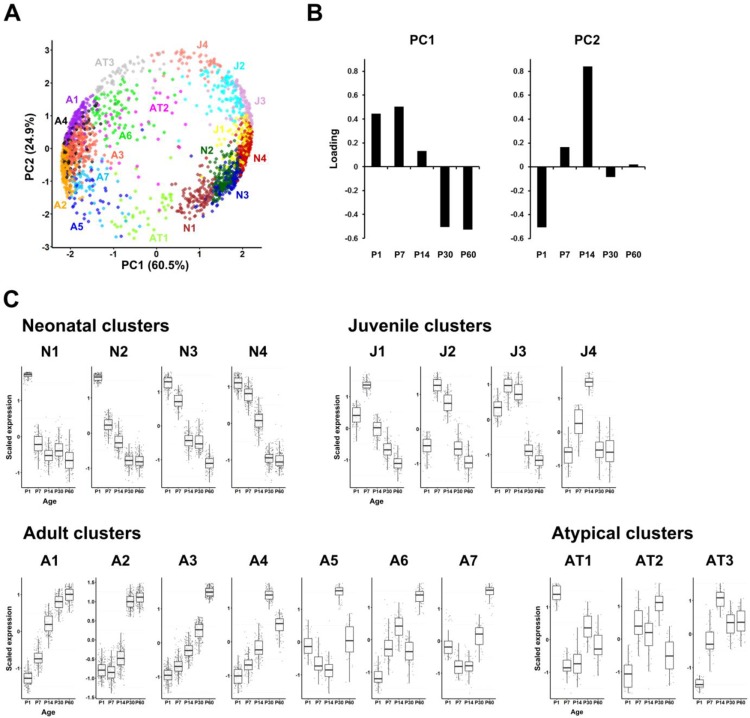
Clustering gene expression patterns during postnatal heart development in control mice. (A) PCA with SOM clustering of gene expression. The expression profile of each gene is represented, and genes belonging to different clusters are indicated by different colors and separated by PC1 and PC2. (B) Loading plots for PC1 and PC2 showing contributions of each variable (developmental stage) to the primary components. (C) Expression profiles of each SOM cluster. Scaled expression along developmental stages is shown in box plots together with cluster categories and names (Neonatal clusters: N1-N4; Juvenile clusters: J1-J4; Adult clusters, A1-A7; Atypical clusters: AT1-AT3).

To determine temporal transcriptional changes occurring during postnatal heart development of normal mice at the organ level, we conducted transcriptome analysis using microarray data of cardiac ventricle samples obtained from control (MCK-Cre) mice of ages P1, P7, P14, P30, and P60. We chose these ages because these would cover all the important events such as a shift in the mode of cardiomyocyte growth from hyperplasia to hypertrophy during the course of postnatal heart development. First, principal component analysis (PCA) was performed to identify the sources of variance in temporal mRNA expression changes detected by microarray analysis (S4 Fig). We used a data set consisting of genes within the top 30% coefficient of variation (CV) (2035 genes in total) in order to focus on the genes whose expression dynamically changed during postnatal heart development. PCA revealed that the first two components, PC1 and PC2, explained ~85% of the variance (Fig 1A). PC1, explaining 60.5% of total variance, represented differential gene expression between early (P1, P7) and late (P30, P60) postnatal developmental stages (Fig 1B). PC2, explaining 24.9% of total variance, represented a sequentially changing pattern during early (P1-P14) postnatal heart development (Fig 1B). These data indicate that, though the majority of changes in gene expression occur in first two weeks after birth, transcriptional changes leading to functional maturation of the adult heart still continue at 1–2 months after birth.

To organize the transcriptome of postnatal heart development into functional clusters, we constructed self-organizing maps (SOMs) which generate clusters with a network topology reminiscent of biological systems [30] (S4 Fig). To construct SOMs from our data, we started from two clusters, since the PCA score plot suggested the minimum of two gene clusters along the PC1 axis (Fig 1A). Subsequent analyses concluded that 18 clusters with a 3 X 6 configuration were the most adequate in terms of separation of distinct expression patterns (Fig 1C) and gene ontology (GO) terms (see below). These 18 clusters could be sub-categorized into 4 classes (neonatal, juvenile, adult, and atypical) based on their characteristic expression patterns (Fig 1A and 1C): genes in neonatal clusters (N1-N4) whose expression is highest at P1 and then continually decreases afterwards, genes in juvenile clusters (J1-J4) whose expression is highest at P7 or P14, and genes in adult clusters (A1-A7) whose expression stays low during neonatal and juvenile stages and exhibits a major increase towards adult stages at P30 and/or P60. Three clusters, which did not exhibit stage-specific expression patterns, were classified as atypical (AT1-AT3). Clear demarcation of these expression patterns, neonatal, juvenile, and adult, suggests that the genes classified into different clusters likely play specific roles during each developmental window, achieving functional adjustments as the postnatal heart matures.

To test this idea, GO term enrichment analysis was performed for each gene cluster represented in S4 Fig As summarized in Table 1, GO terms enriched for neonatal, juvenile, and adult class of clusters demonstrated functions relevant to the corresponding stages of postnatal heart development (see S2 Table for the full lists of GO terms). For example, the genes in the neonatal clusters (N1-N4) are enriched for the GO terms related to heart development, heart contraction, cell proliferation, RNA splicing, RNA transport, translation, and actin cytoskeleton. Among these terms, cell proliferation [6, 51, 52] and splicing (alternative splicing of the developmentally regulated genes) [10] are known to play critical roles in the maturation of the postnatal heart. After birth, the growth of the heart shifts from cell proliferation to cellular hypertrophy and alternative splicing is utilized in fetal to adult isoform switching. Accordingly, the neonatal cluster genes reflecting these GO terms have their highest expression at the neonatal stages and rapidly decline afterwards. *Nkx2-5* [53] and *Myh7* [54] which are known to be essential for embryonic heart development were also classified in the neonatal cluster (N1). These results validate biological relevance of the clusters identified by the current analysis using SOMs.

**Table 1:** Characteristic GO terms enriched in the SOM clusters.

Cluster	# of genes	Category	GO term (*p*-value)
N1	121	Neonatal	heart development (2.8X10^-3^), regulation of heart contraction (6.6X10^-3^), mRNA transport (7.0X10^-3^)
N2	186	Neonatal	cell cycle (1.0X10^-7^), RNA splicing (2.2X10^-5^), cell division (2.4X10^-4^), nuclear division (4.3X10^-3^), organelle fission (1.2X10^-3^)
N3	149	Neonatal	ribosome (3.0X10^-13^), translation (4.5X10^-8^)
N4	207	Neonatal	actin cytoskeleton (3.6X10^-4^), RNA splicing (3.8X10^-3^), chromosome organization (6.7X10^-3^), spliceosomal complex (7.3X10^-3^)
J1	108	Juvenile	actin cytoskeleton (2.5X10^-9^), cytoskeleton organization (2.0X10^-7^), cell junction (2.5X10^-3^)
J2	92	Juvenile	cell communication (1.5X10^-4^), extracellular matrix (3.9X10^-4^), response to stimulus (1.2X10^-3^), cell adhesion (2.9X10^-3^), blood vessel development (3.8X10^-3^)
J3	95	Juvenile	cell adhesion (3.1X10^-5^), blood vessel development (1.4X10^-4^), extracellular matrix (6.9X10^-3^), cell migration (8.3X10^-3^)
J4	52	Juvenile	cell differentiation (6.2X10^-6^), apoptotic process (3.0X10^-4^), vasculature development (3.8X10^-4^), immune system process (8.3X10^-4^), regulation of cell cycle (9.2X10^-4^)
A1	165	Adult	mitochondrion (3.5X10^-9^), contractile fiber (5.3X10^-3^)
A2	211	Adult	mitochondrion (1.9X10^-17^), cofactor metabolic process (2.0X10^-3^), fatty acid metabolic process (3.0X10^-3^), cellular amino acid metabolic process (2.8X10^-3^)
A3	233	Adult	mitochondrion (7.0X10^-14^), contractile fiber (3.0X10^-5^), cofactor metabolic process (9.1X10^-4^), transport (5.2X10^-3^)
A4	122	Adult	mitochondrion (7.5X10^-3^)
A5	40	Adult	n.e.
A6	78	Adult	mitochondrion (5.4X10^-6^), response to starvation (8.5X10^-4^), oxidation-reduction process (7.2X10^-3^)
A7	49	Adult	n.e.
AT1	38	Atypical	n.e.
AT2	34	Atypical	extracellular matrix (6.6X10^-4^)
AT3	55	Atypical	n.e.

GO terms representing specific biological processes or cellular components were shown up to 5 terms per cluster. n.e.: no enrichment of specific GO term.

The GO terms enriched for the juvenile clusters (J1-J4) include blood vessel/vasculature development and related biological processes such as cell adhesion and migration (Table 1). This result indicates that vascularization of the heart is most active at 7 to 14 days after birth in mice. Indeed, it has been reported that intense capillary growth in the heart occurs during this period [55]. Genes involved in actin cytoskeleton are also enriched in the juvenile cluster J1, suggesting that the dynamic reorganization of cytoskeletal assembly in the heart is still ongoing during the early juvenile stage of postnatal heart development.

The GO terms enriched in the adult clusters A1-4 and 6 include contractile fibers as well as mitochondrial metabolic processes (e.g. fatty acid metabolic process). The biological functions represented by these terms, fatty acid metabolism and contractility, are essential for adult cardiac function. Mitochondrial numbers increase as the heart grows and structural and functional maturation of mitochondria and myofibrils in cardiomyocytes accelerates during the postnatal development of the heart [56]. Therefore, the genes classified to these adult cluster genes epitomize the physiological changes transforming the heart into the mature adult state.

### Identification of hub genes of each developmental cluster

The GO analysis described above revealed distinct stage-specific gene clusters with biologically relevant functions corresponding to each stage of postnatal heart development (Table 1). Since the failing heart exhibits misregulated expression of genes specific to fetal and adult developmental periods [12, 13, 57], identifying key genes in the developmentally classified clusters may provide useful biomarkers in clinical applications. To identify candidate biomarkers, we next conducted gene co-expression network analysis to gain further insights into the progression of postnatal heart development at the individual gene level (S4 Fig). Gene co-expression network analysis is a powerful approach which deduces regulatory networks of genes conceivably related in functions based on the similarity (co-expression) in their expression patterns [58, 59]. In particular, highly connected genes (hub genes) in gene co-expression networks have been shown to play critical roles in diverse cell types as well as pathophysiological conditions [60–67]. Among currently available gene co-expression network analyses, the weighted correlation network analysis (WGCNA) package has many advantages, enabling us to determine the threshold in a data-driven way (using the scale-free topology criterion), to convert the co-expression measure to a connection weight (by soft-thresholding), and to measure connectivity with high biological signals (using the topological overlap matrix) [31]. However, since network construction is usually highly sensitive to noise which most expression data have, a single application of WGCNA is unlikely to produce robust and reproducible results, and therefore it is hard to assess the significance of hub genes. To address these issues, we incorporated a bootstrap inference [32] into WGCNA and ranked highly connected hub genes based on the average connectivity score across networks built on bootstrapped replications of the data (S3 Fig). We applied this method to a gene set of each SOM cluster to identify robust hub genes that are potentially regulating functional maturation of the postnatal heart.

Hub genes identified in each cluster following this method are summarized in Table 2 (see S3 Table for full lists). As expected, the high-ranking hub genes identified for the neonatal, juvenile and adult clusters demonstrated shared biological functions with the GO terms enriched in each cluster. A significant number of neonatal cluster hub genes are implicated in cell proliferation: *Rbmx* [68] and *Igf2bp2* [69, 70] in cluster N1, *Cenpt* and *Cenpw* [71] in cluster N2, *Mcm5* [72] in cluster N3, and *Dstn* [73] in cluster N4. *Rbmx* [74] in cluster N1 and *Sf3a3* [75] in cluster N3 are implicated in mRNA splicing. As described earlier, these GO terms such as cell proliferation and alternative splicing are significantly enriched in the neonatal clusters (Table 1). Additionally, genes involved in apoptosis (*Ngfrap1* [76] in cluster N2) and glycolysis (*Eno1* [77] in cluster N1) were identified as hub genes in neonatal clusters. Though cardiomyocyte proliferation [6, 51, 52], apoptosis of cardiomyocytes [51], alternative splicing [10], and glycolysis [8] are all highly active during the fetal to neonatal stages, the above hub genes implicated in these biological functions have not been investigated in the context of postnatal heart development. Therefore, these genes may hold yet unexplored mechanisms for the functional switch from the fetal to adult type heart. Another interesting finding is that pro-hypertrophic (*H2afz* [78]) and anti-hypertrophic (*Grb10* [79]) genes were identified as hub genes in the clusters N3 and N2, respectively, suggesting that hypertrophic growth of the postnatal heart may be regulated by the balance of pro- and anti- hypertrophy genes.

**Table 2:** Hub genes in the SOM clusters.

Cluster	Hub gene	Known/potential function in the heart
N1	*Eno1*^**1**^, *Rbmx*^**2,3**^, *Gm16379*, *Ears2*, *Msmo1*, *Igf2bp2*^**2**^	**1** glycolysis, **2** cell proliferation, **3** mRNA splicing
N2	*Armcx4*, *Aldh1b1*, *Cenpt*^**1**^, *Tubb2b*, *Ngfrap1*^**2**^, *Grb10*^**3**^, *Armcx2*, *Cenpw*^**1**^, *Cdkn1c*^**4**^, *Sept6*	**1** cell cycle, **2** apoptosis, **3** anti-hypertrophy, **4** inhibition of cell cycle
N3	*Col18a1*^**1**^, *Ndn*, *Sf3a3*^**2**^, *Sox12*, *Mcm5*^**3**^, *Vim*, *Prdx4*, *H2afz*^**4**^	**1** anti-angiogenesis, **2** mRNA splicing, **3** DNA replication, **4** hypertrophy
N4	*Eef1a1*, *Dstn*^**1**^, *Lpar4*^**2**^, *Gm6548*, *Ramp1*^**2**^, *Hist1h2ae*, *Chsy1*, *Bhlhb9*, *Col5a1*^**3**^, *Actr3*, *Tspan6*^**4**^	**1** cell migration and proliferation, **2** angiogenesis, **3** heart valve development, **4** inhibition of innate immune response
J1	*Ubtd2*, *Cmtm3*, Arpc5, Adam19^**1**^, *Nras*, *Evl*	**1** heart valve and septum development
J2	*Rnf144a*^**1**^, *Esam*^**2**^, *Galnt16*, *Ets2*^**2**^, *Chst1*	**1** cell migration, **2** angiogenesis
J3	*Nrep*^**1**^, *Prcp*^**2**^, *Fmnl3*^**2**^, *Dusp6*^**3**^, *Lasp1*^**1**^	**1** cell migration, **2** angiogenesis, **3** inhibition of cardiomyocyte proliferation
J4	*Bcl6b*^**1**^, *Robo4*^**1**^, *Egr1*^**1**^	**1** angiogenesis
A1	*Rpl3l*, *Ckmt2*, *Dnajc21*, *Sdr39u1*, *Hspb8*^**1**^, *Myl12a*, *Mvp*, *Eif4e3*, *Cebpg*	**1** activator of nuclear and mitochondrial functions of STAT3
A2	*Phyhd1*, *Gstk1*^**1**^, *Dnajc28*, *Txlnb*, *Scube2*^**2**^, *Zfp106*, *Oma1*^**3**^, *Slc4a3*, *Ppm1k*^**4**^, *Pxmp2*^**5**^, *Pcca*^**6**^	**1** detoxification of xenobiotic compounds and lipid peroxide products, **2** maintenance of the adult coronary vasculature, **3** mitochondrial quality control, **4** regulation of mitochondrial membrane permeability transition pore opening and regulation of Bckdha activity, **5** peroxisomal channel, **6** catabolism of branched-chain amino acids and fatty acids
A3	*Tango2*, *Xirp2*^**1**^, *Lpl*^**2**^, *Il15*^*3*^, *Cmya5*^**4**^, *Hrc*^**5**^, *Coq10a*, *Klhdc1*, *Rab11fip3*, *Bckdha*^**6**^, *Cpeb3*, *Pkia*^**7**^	**1** intercalated disc maturation, **2** lipid metabolism, **3** protection from oxidative stress, **4** myofibrillogenesis, **5** calcium homeostasis, **6** branched-chain amino acid metabolism, **7** regulation of heartbeat
A4	*Psme4*^**1**^, *Prodh*^**2**^, *Ank1*, *Lrg1*^**3**^, *Chpt1*, *Lyz1*^**4**^, *Mavs*^**4**^	**1** maintenance of mitochondrial function, **2** protection from oxidative stress, **3** angiogenesis, **4** innate immune response
A5	*Hist1h2bk*^**1**^, *Dpep1*	**1** plasminogen receptor
A6	*Acadm*^**1**^, *Snrpn*^**2**^, *Klf9*, *Pex11a*^**3**^	**1** fatty acid β-oxidation, **2** regulation of mitochondrial gene expression, **3** regulation of peroxisome elongation and division
A7	n.d.	
AT1	*Ppm1g*^**1**^, *Ap2a1*	**1** pre-mRNA splicing
AT2	n.d.	
AT3	*Asph*^**1**^, *Dysf*^**2**^, *Slc44a2*	**1** regulation of SR Ca release and contractility, **2** cardiomyocyte membrane repair

Hub genes ranked in top 5% are shown. In clusters A7 and AT2, hub gene was not able to be determined because the scale-free topology criterion was not satisfied in networks of these clusters (see text for details). n.d.: not determined.

In the juvenile clusters, hub genes whose biological functions are implicated in angiogenesis dominated (Table 2): *Esam* [80] and *Ets2* [81] in cluster J2, *Prcp* [82] and *Fmnl3* [83] in cluster J3, *Bcl6b* [84], *Robo4* [85], and *Egr1* [86] in cluster J4. In addition to the angiogenesis-related genes, a gene involved in heart valve and septum development (*Adam19* [87] in cluster J1) was also identified as a juvenile cluster hub gene. Interestingly, a hub gene in cluster J3 (*Dusp6*) is reported to have an inhibitory effect in cardiomyocyte proliferation [88], suggesting that this gene could be a key negative regulator of cardiomyocyte proliferation.

Many of the hub genes identified in the adult gene clusters (A1-A7; Table 2) encode genes involved in metabolism, including mitochondrial enzymes and coenzymes for lipid metabolism (*Pcca* [89] in cluster A2, *Lpl* [90] in cluster A3 and *Acadm* [91] in cluster A6) and amino acid metabolism (*Pcca* [89] in cluster A2 and *Bckdha* [92] in cluster A3). Also related to mitochondrial function, the following are identified as hub genes: protection of mitochondria from oxidative stress (*Gstk1* [93] in cluster A2 and *Prodh* [94] in cluster A4), regulating mitochondrial gene expression (*Snrpn* [95] in cluster A6), and regulating/maintaining mitochondrial functions (*Hspb8* [96] in cluster A1, *Oma1* [97] and *Ppm1k* [98, 99] in cluster A2, and *Psme4* [100] in cluster A4). The adult cluster hub genes described above not only confirm that metabolic conversion is essential for the functional maturation of the postnatal heart, but also identify for the first time the temporal dynamics of this conversion (Fig 1C).

The genes in adult clusters A4 and A5 exhibited the highest expression level at one month after birth instead of two months as the other adult clusters (Fig 1C). Interestingly, some of the hub genes in these two clusters have functions in innate immune response (*Lyz1* [101] and *Mavs* [102] in cluster A4, and *Hist1h2bk* [103] in cluster A5) (Table 2), suggesting that innate immune response may be necessary for the functional maturation of the young adult heart. Cluster A4 also contains a hub gene which is involved in angiogenesis (*Lrg1* [104]). This is quite intriguing because the rest of angiogenesis-related hub genes were found in the juvenile clusters. In addition, one of the hub genes in cluster A2, *Scube2*, is also implicated in maintenance of the adult coronary vasculature [105, 106]. Therefore, these two blood vessel-related genes may play an important role in the coronary vessel maintenance in the adult heart.

### Identification of differentially expressed (DE) genes reveals a potential role of Sox6 in the suppression of cell proliferation in the postnatal heart

We have previously reported that Sox6 likely plays a role in postnatal heart function [18]. Mice homozygous for a Sox6 null mutation die within two weeks after birth and presented cardiac arrhythmia [18]. To investigate the function of Sox6 in postnatal heart development, we determined differentially expressed (DE) genes between the Sox6 KO (Sox6^loxp/loxp^; MCK-Cre), in which Sox6 is genetically ablated in both skeletal muscle and heart, and the control mice (MCK-Cre) hearts during postnatal development. The inactivation of Sox6 by MCK-Cre in the cardiac muscle did not show any noticeable change in gross morphology in the heart (data not shown). Reduction of *Sox6* mRNA levels in the postnatal heart of Sox6 KO mice were confirmed by RT-qPCR (S5 Fig). Differential gene expression analysis and GO term enrichment analysis revealed substantial number of DE genes and associated GO terms at each postnatal age (Table 3; see S4 Table for a full list of DE genes). Further analysis indicated that the Sox6 binding motif is significantly enriched in the promoter regions of upregulated genes at P7, P30, and P60 as well as downregulated genes at P14 and P30 (S6 Fig), suggesting possible direct regulation of many of the DE genes by Sox6 at these postnatal ages in the murine heart. In these DE genes at P7, genes associated with GO terms for cell proliferation such as cell cycle, cell division, organelle fission, nuclear division, and mitosis (e.g. *Ccnb1*, *Ccnd1*, *Cdc20*, *Cdca8*, and *Cdk1*) were significantly upregulated in the Sox6 KO ventricle. Since cardiomyocyte proliferation ceases a few days after birth [6, 51, 52], the high expression of genes involved in cell proliferation in the Sox6 KO heart suggested that cell division and/or nuclear division might be still active at P7 in the absence of Sox6 function.

**Table 3:** DE genes between control and Sox6 KO hearts.

Age	Category	# of DE probes	GO term (*p*-value)
P1	KO>Control	30	protein complex subunit organization (7.9X10^-3^)
	Control>KO	16	mitochondrion (5.3X10^-4^)
P7	KO>Control	147	cell cycle (6.5X10^-10^), cell division (2.7X10^-9^), organelle fission (3.5X10^-9^), nuclear division (1.2X10^-8^), mitosis (1.2X10^-8^)
	Control>KO	93	antigen processing and presentation (8.9X10^-4^)
P14	KO>Control	75	n.e.
	Control>KO	43	programmed cell death (2.2X10^-3^), regulation of cell division (7.7X10^-3^)
P30	KO>Control	109	sarcomere (2.4X10^-3^), cell differentiation (3.0X10^-3^)
	Control>KO	76	extracellular matrix (1.9X10^-6^), extracellular region (4.5X10^-5^), basement membrane (4.7X10^-4^)
P60	KO>Control	59	n.e.
	Control>KO	39	n.e.

GO terms representing specific biological processes or cellular components were shown up to 5 terms per category. n.e.: no enrichment of specific GO term.

**Fig 2:**
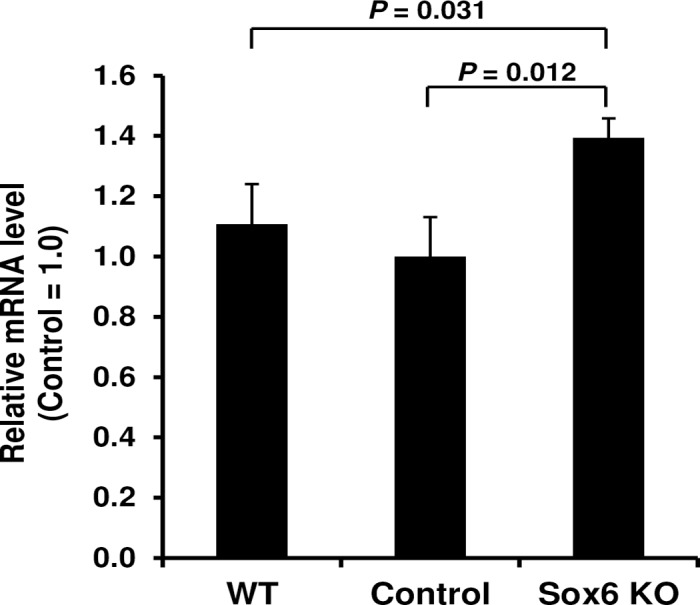
Cyclin D1 is upregulated in the Sox6 KO heart at postnatal day 7. Total RNA was extracted from ventricles of wild type (WT), control and Sox6 KO mice at postnatal day 7 (P7), and the mRNA level of cyclin D1 (*Ccnd1*) was quantified by RT-qPCR. Data are normalized for those from control mice and represented as mean ± s.d. (*n* = 3).

**Fig 3:**
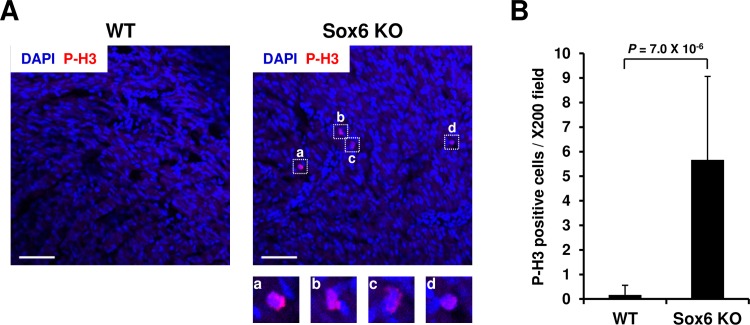
Sox6 KO mice still contain proliferating cells in the ventricle at postnatal day 7. (A) Representative views of immunofluorescence images from wild type (WT) and Sox6KO (Sox6^f/f^; MCK-Cre) ventricles. Cryosections of postnatal day 7 (P7) ventricles from WT and Sox6KO mice were stained with antibody against phosphorylated histone H3 (P-H3) (red) and DAPI (blue). Magnified views of P-H3 positive cells (a-d) were also shown for the Sox6 KO cryosection. Scale bars: 50 μm. (B) Quantification of P-H3 positive cells. P-H3 positive cells were counted on WT and Sox6 KO immunofluorescence images (12 images each) and represented as mean ± s.d. per X200 field covering approx. 0.4 mm^2^ (630 μm X 630 μm).

To test this hypothesis, we compared the mRNA levels of a well-defined marker for active cell cycle, cyclin D1 (*Ccnd1*) [107], between Sox6 KO, control and wild type (WT) hearts at P7. We chose *Ccnd1* among a number of cell cycle-related genes because it is implicated as a direct target of Sox6 [23, 108] and is found to be differentially expressed in our microarray dataset (S4 Table). As shown in Fig 2, *Ccnd1* mRNA level was significantly higher at P7 in the Sox6 KO heart compared to WT and control hearts, corroborating our microarray result. Our result also suggests that the control (MCK-Cre) heart and wild type (C57BL/6) heart may have no difference in terms of cell cycle progression in the early postnatal heart. These results indicate that, as suggested by the increased transcription of genes promoting cell proliferation, cell cycle progression is still occurring as late as P7 in the Sox6 KO heart. This led us to investigate whether the Sox6 KO heart presents cell proliferation at P7 at which normally hyperplasic growth is already ceased and hypertrophic growth is dominant in the heart. We performed phospho-histone H3 (P-H3) staining, which specifically marks any type of cells in the cell cycle phases between G2/M and anaphase [109, 110], using the ventricular myocardium of Sox6 KO mice and wild type mice. As shown in Fig 3, the loss of Sox6 protein caused a significant increase in the number of stained cells. Namely, wild type heart exhibited no discernable P-H3 staining at P7, while the Sox6 KO heart exhibited, albeit at a low level, P-H3 positive cells consistently (Fig 3). These results suggest that at P7 in the Sox6 KO heart, more mitotic cells exist compared to the wild type heart, and therefore that Sox6 possibly has a suppressive role in cell cycle progression in the early postnatal heart.

### DE hub genes in the Sox6 KO heart predict additional roles of Sox6 in postnatal heart development

**Fig 4:**
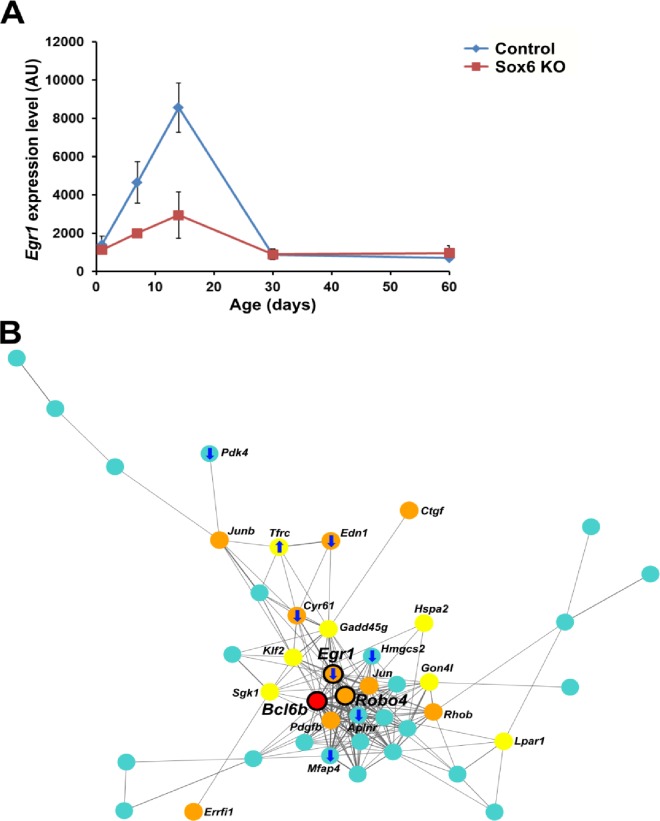
*Egr1* is downregulated in the Sox6 KO heart. (A) Differential expression of *Egr1* in control and Sox6 KO hearts revealed by our microarray analysis. AU: arbitrary unit. (B) A weighted gene co-expression network of the cluster J4. A core network module with a 0.05 minimum connectivity is demonstrated together with information on gene functions (GO terms) and gene expression. Genes related to vasculature development (GO:0001944), cell differentiation (GO:0030154) and both are shown in red, yellow, and orange, respectively, while other genes are shown in sky blue. Identified hub genes are indicated by black outlines with larger labels. One of the hub genes (*Bcl6b*) is also shown in red because it is known to be involved in angiogenesis (see text). The width of edges (lines between genes) reflects the strength of TOM-based connectivity between genes (i.e. the degree of co-expression). Directions of expression change at P7 and/or P14 in the Sox6 KO heart are indicated by arrows.

Functional annotation of the Sox6 KO heart transcriptomes identified GO terms significantly enriched in the DE genes between the Sox6 and control hearts (Table 3). To better infer possible functions of Sox6 in postnatal heart development, we focused our analysis on the hub genes identified for the control heart (Table 2) and compared their expression levels with those in the Sox6 KO heart. It was found that six hub genes identified for the control heart are differentially expressed in Sox6 KO heart (Table 4). Among these, *Egr1* in cluster J4 (Table 2), which is involved in angiogenesis [86], cardiac hypertrophy [111], and cardioprotection [112], demonstrated significantly decreased expression at P7 and P14 in the Sox6 KO hearts compared to control (Fig 4A). Since expression of genes promoting angiogenesis was significantly upregulated during the juvenile stage (Tables 1 and 2), the reduced expression of *Egr1* may have a functional consequence. To further investigate the behavior of juvenile cluster angiogenesis genes in the Sox6 KO heart, we mapped information of GO terms and changes in gene expression detected in the Sox6 KO heart on a gene co-expression network of cluster J4. Genes related to the GO terms vasculature development and cell differentiation (*Cyr61*, *Edn1*, and *Egr1*) were downregulated in the Sox6 KO heart (Fig 4B), suggesting that the loss of Sox6 in the heart might delay maturation of blood vessels due to the reduced expression level of *Egr1*. Our data is in agreement with the report showing that overexpression of miR-499, which targets Sox6 and reduces its expression, resulted in a decrease in the *Egr1* transcript level [113]. Together, these observations suggest that Sox6 may positively regulate the expression of *Egr1* to regulate maturation of blood vessels in the postnatal heart.

**Table 4:** DE hub genes in the Sox6 KO heart.

Gene	Cluster	Fold change in Sox6 KO	Known/potential function in the heart
*Tubb2b*	N2	1.9 (P7)	Unknown
*Egr1*	J4	0.4 (P7), 0.3 (P14)	Angiogenesis, hypertrophy, cardioprotection
*Hspb8*	A1	0.6 (P30)	activator of nuclear and mitochondrial functions of STAT3
*Il15*	A3	0.6 (P7)	Protection from oxidative stress
*Lyz1*	A4	0.7 (P7), 0.6 (P30)	innate immune response
*Klf9*	A6	2.5 (P1)	Unknown

DE hub genes were also found in the adult clusters. For example, the expression level of *Il15* in adult cluster A3 was found to be decreased at P7 in the Sox6 KO heart (Table 4). It has been recently demonstrated that *Il15* protects cardiomyocytes from oxidative stress [114], suggesting that *Il15* could be involved in protection from oxidative stress during postnatal heart development, and Sox6 could be a positive regulator of *Il15* in the early postnatal heart. *Lyz1* in adult cluster A4 was also differentially expressed at two time points (decreased at P7 and P30) in the Sox6 KO heart (Table 4). This gene encodes an antimicrobial enzyme [101] and is an important component of innate immune system [115], suggesting that Sox6 could be also involved in innate immune system in the developing postnatal heart.

## Discussion

**Fig 5:**
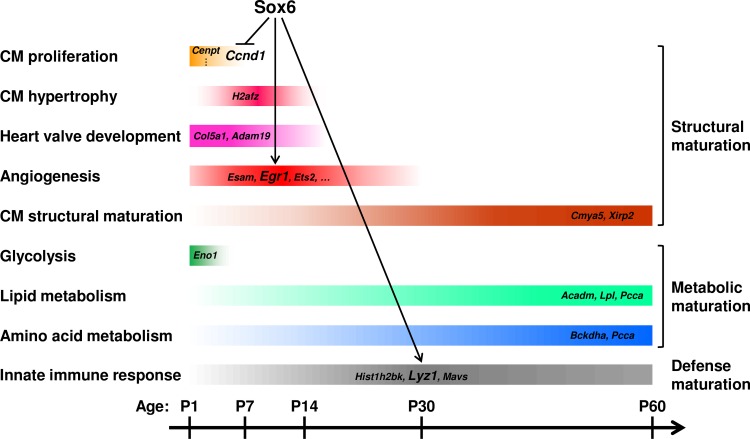
Schematic representation of key events and inferred roles of Sox6 during postnatal heart development in mice. Temporal changes of key events during postnatal heart development in control mice are demonstrated together with associated hub genes. Inferred roles and potential targets of Sox6 are also shown with arrows and larger labels. CM: cardiomyocyte.

The heart undergoes dramatic changes after birth, including transformations in modalities of tissue growth, contractility, and metabolic properties. Understanding the molecular mechanisms of the functional maturation of the postnatal heart could have important clinical implications, because the failing heart is known to demonstrate the reversal in the expression of contractile protein isoforms and metabolic enzymes from an adult to fetal/neonatal state [12, 13]. Therefore, systematic and statistically robust analysis of longitudinal gene expression of the postnatal heart development at the organ level would contribute to our understanding of the molecular mechanisms of heart failure. To obtain this information, we developed a bioinformatics pipeline integrating SOM clustering and gene co-expression network analysis with bootstrap inference. Using this newly developed method, we successfully identified distinct co-expressed gene clusters in the developing postnatal heart and statistically robust hub genes in developmentally classified clusters. The gene clusters reported here are enriched with functionally relevant genes to each developmental window of the postnatal heart, and many of these genes have not been implicated in heart development before. Furthermore, the DE gene analysis between the Sox6 KO and control hearts uncovered previously unknown Sox6 functions in postnatal heart development, namely suppression of cell proliferation and upregulation of angiogenesis genes in the postnatal heart (Fig 5).

18 gene clusters classified through SOM clustering were categorized into 4 groups, neonatal, juvenile, adult, and atypical, based on their temporal expression patterns. The neonatal clusters (N1-N4), which have the highest expression at P1, were enriched with genes involved in cell proliferation. This observation matched well with the fact that cell proliferation is active in the neonatal heart and ceases shortly thereafter. Since restoring the proliferative activity of cardiomyocytes in the adult heart is of great interest in regenerative medicine [116], the cell proliferation-related hub genes identified in the neonatal clusters (*Rbmx*, *Igf2bp2*, *Cenpt*, *Cenpw*, *Mcm*5, and *Dstn*) are candidate targets for restoring proliferative activity of cardiomyocytes in the adult heart. The glycolytic enzyme Eno1 (an N1 cluster hub gene: Table 2) is also reported to have a positive effect on cell proliferation in a hepatocellular carcinoma cell line [117], suggesting that *Eno1* may be another key regulator of cardiomyocyte proliferation. In the Sox6 KO heart, expression of these hub genes of the neonatal clusters was not altered (data not shown). However, GO term analysis of upregulated genes at P7 in the Sox6 KO heart identified significant enrichment of the genes playing roles in cell proliferation and cell cycle progression (Table 3). This result suggested a possible role of Sox6 in cell proliferation. As expected, we observed increased expression of the cell cycle marker gene *Ccnd1* in the Sox6 KO heart (Fig 2), and also detected phosphorylated histone H3-positive cells in the Sox6 KO ventricle at P7 which were not seen in the wild type heart (Fig 3). These observations suggest that, although our immunohistochemical analysis does not distinguish the types of stained cells, Sox6 likely plays an inhibitory role in cell cycle progression. It is well documented that the mode of cardiac growth switches from hyperplasia (increase in cell number) to hypertrophy (increase in cell volume) soon after birth with multinucleation and polyploidization, which contributes to the postnatal growth of the heart [5–7]. Therefore, the prolonged cell proliferation observed in the Sox6 KO ventricular myocardium suggests that Sox6 promotes the switch from the fetal to adult mode of cardiac growth, and therefore, the loss of a functional Sox6 protein results in delaying the cessation of cell cycle progression. In light of this, the upregulation of *Nppa* (ANP) and *Nppb* (BNP) at P7 in the Sox6 KO ventricle (S4 Table), which are considered to be fetal type genes in the heart [12, 118], further provides support for our hypothesis that Sox6 promotes the switch from the fetal to adult state of the heart. Although the current study cannot rule out potential indirect effect of *Sox6* knockout in skeletal muscle and our immunohistochemical data does not distinguish the cell types of mitotic cells and a degree of cytokinesis, future study characterizing the cell types of mitotically active cells, distinct cell cycle phases, cytokinesis as well as their quantification using heart-specific KO mice (e.g. using Tnnt2-Cre mice) would facilitate our understating of more precise role of Sox6 during postnatal heart development.

Another interesting finding of our study is that the juvenile gene clusters were enriched with genes involved in angiogenesis (Table 1), indicating active growth of blood vessels at P7-P14 in the postnatal heart. This observation matched well with a previous report showing that intense capillary growth occurs during juvenile stages [55]. To our knowledge, our current report is the first demonstration of highly active transcription of a large set of angiogenesis-related genes (e.g. *Egr1*) at juvenile stage in the postnatal heart. Because control of the angiogenic process is crucial for regeneration and repair of the adult heart, the newly identified angiogenesis hub genes in this report could be clinically relevant. Given that Sox6 appears to be involved in transcriptional upregulation of crucial angiogenic genes including *Egr1* in the postnatal heart (Fig 4 and Fig 5)[113], further investigation of the role of Sox6 in angiogenesis in the postnatal heart, especially focusing on cellular communication between cardiac myocytes and non-muscle cells (such as endothelial cells), could identify promising targets for rebuilding blood vessels to repair the damaged heart.

In our analysis of DE genes between the Sox6 KO and control hearts, the majority of genes showed differential expression only at a single time point, while some genes demonstrated consistent differential expression at multiple time points (S7 Fig). Of these, at all ages tested, 9 genes were consistently upregulated in Sox6 KO [hemoglobin beta adult major chain (*Hbb-b1*), myocardin (*Myocd*), post-GPI attachment to proteins 2 (*Pgap2*), phosphatidylinositol binding clathrin assembly protein (*Picalm*), proteasome 26S subunit 8 (*Psmd8*), retinitis pigmentosa GTPase regulator interacting protein 1 (*Rpgrip1*), sterile alpha motif domain containing 4 (*Samd4*), ubiquitin-conjugating enzyme E2J 2 (*Ube2j2*), zinc finger with KRAB and SCAN domains 17(*Zkscan17*)], while 2 genes were consistently downregulated in Sox6 KO heart [leucine carboxyl methyltransferase 1 (*Lcmt1*) and nudix-type motif 6 (*Nudt6*)]. Among these, *Myocd* is known to be involved in cardiovascular development [119] as well as maintenance of heart function [120]; therefore, the current result may indicate that *Myocd* also plays an important role in the functional maturation of the postnatal heart. Although the functions of the rest of the genes in heart development are as yet unknown, our result indicates that they are Sox6 target candidates, thus could be involved in the maturation of the postnatal heart as well.

Recently, O’Meara et al. have performed elegant transcriptomic analysis using RNA-seq characterizing a core transcriptional signature of cardiac myocytes isolated from developing postnatal heart [29]. Our microarray study with the whole cardiac ventricle tissue was able to capture the similar changes in gene expression to that of isolated cardiac myocytes, for instance, upregulation of mitochondrion-related genes and downregulation of cell cycle genes during postnatal heart development (Fig 1 and Table 1). In addition, genes showing distinct expression profiles between the whole cardiac ventricle and isolated cardiomyocytes were also found when our tissue-derived data and the isolated cardiomyocyte-derived data [29] were compared (S8 Fig and S5 Table). Our tissue-derived data contain mixed population-derived gene expression data and this type of comparison would be useful for identifying gene expression profiles specifically contributed by cardiomyocytes and the expression profiles contributed by other cell types in the heart. Of note, *Egr1*, a hub gene in cluster J4, showed clearly different expression patterns between cardiac ventricle tissues and isolated cardiomyocytes (S8 Fig). Since *Egr1* is known to be involved in cardiac hypertrophy [111] and cardioprotection [112] in addition to angiogenesis in the heart [121], the difference in expression patterns may reflect distinct roles in different cell types (e.g. angiogenesis in endothelial cells and hypertrophy/cardioprotection in cardiomyocytes) in the heart. Thus, integrating organ-level and cellular-level data would provide all-inclusive analysis of postnatal heart development.

## Conclusions

We have identified postnatal stage-specific and functionally co-expressed gene clusters that are potential key genes for postnatal heart development. Our newly developed bioinformatics approach is broadly applicable for analyzing time series transcriptome data to discover biologically meaningful results. We have also identified that Sox6 plays an important role in suppressing cell proliferation and modulating fetal gene expression during postnatal heart development. Together, our results provide a framework for organizing the developmental processes of the postnatal heart.

## Supporting Information

S1 Fig Validation of microarray data quality. (A) A box plot showing normalized microarray data from triplicate samples at each developmental stage. (B) A multidimensional scaling (MDS) plot demonstrating similarity between each genotype and each developmental stage. Each axis represents an arbitrary unit and is therefore dimensionless.
(TIF)

S2 Fig Clustering gene expression patterns during postnatal heart development in control mice using mean intensity values. (A) PCA with SOM clustering of gene expression. The expression profile of each gene is represented, and genes that belong to different clusters are indicated by different colors and separated by PC1 and PC2. (B) Loading plots for PC1 and PC2 showing contributions of each variable (developmental stage) to the primary components. (C) Expression profiles of each SOM cluster. Scaled expression along developmental stages is shown in box plots together with cluster categories and names (Neonatal clusters: N1-N5; Juvenile clusters: J1-J4; Adult clusters, A1-A6; Atypical clusters: AT1-AT3). Of note, the SOM clusters obtained with mean intensity values were comparable but slightly different from those with median intensity values (Fig 1), suggesting that there were some variations/noises in intensity values of three biological replicates and they slightly affected clustering of genes.
(TIF)

S3 Fig Degree ranks of hub genes. Genes were sorted according to the average ranking calculated from TOM-based connectivities after bootstrap inference (the more connectivity, the smaller the rank), and plotted against standard deviation (s.d.) of the ranks. Each dot represents individual gene. Genes ranked in top 50% in each SOM cluster are shown. Red dots: hub genes (ranked in top 5%). Black dots: other genes.
(TIF)

S4 Fig Outline of our transcriptomic analysis. Workflow for analyzing control and Sox6 KO mice is shown. Data and results are shown in red, and processing steps are shown in green.
(TIF)

S5 Fig *Sox6* mRNA levels in control and Sox6 KO mice hearts. Total RNA was extracted from control and Sox6 KO mice ventricles, and *Sox6* mRNA levels were quantified by RT-qPCR. Data are normalized for the reference genes (see the Methods section for details) and represented as mean ± s.d. (*n* = 3).
(TIF)

S6 Fig Enrichment of the Sox6 binding motif in the promoter regions of DE genes in Sox6 KO mice. Significant enrichment of the Sox6 binding motif is shown as adjusted *p*-values (highlighted) obtained by AME.
(TIF)

S7 Fig DE genes between control and Sox6 KO hearts. The number of probes for DE genes was counted at each time point and is shown as bar graphs. DE genes unique to each time point are shown in dark gray, and DE genes at multiple time points are shown in color (i.e. the same color indicates the same set of genes). (A) Upregulated genes in the Sox6 KO heart. (B) Downregulated genes in the Sox6 KO heart.
(TIF)

S8 Fig Comparison of gene expression data of this study and those of O’Meara et al. (2015). Expression profiles of three hub genes from different SOM clusters (*Eno1* from cluster N1, *Egr1* from cluster J4, and *Lyz1* from cluster A4) obtained by this study and O’Meara et al. (2015) are exemplified (graphs are generated using S5 Table). As for the data from O’Meara et al. (2015), both tissue (cardiac ventricle)-derived data (vP0, vP4, vP7, and vAd data in S5 Table) and isolated cardiomyocyte (CM)-derived data (iP0, iP4, iP7, and iAd data in S5 Table) are shown. *Eno1* showed similar expression patterns between iCM and cardiac ventricle, suggesting that *Eno*1 expression is similar between CM and other types of cells in cardiac ventricle. In contrast, *Egr1* and *Lyz1* showed distinct expression patterns between iCM and cardiac ventricle, suggesting that expression of *Egr*1 and *Lyz*1 is different between CM and other types of cells in cardiac ventricle.
(TIF)

S1 Table PrimeTime qPCR Assays and TaqMan Gene Expression Assays used in the experiments. (XLSX)

S2 Table Full lists of genes, SOM clusters, and GO terms. (XLSX)

S3 Table Full lists of degree ranks of hub genes. Genes were sorted according to the average ranking calculated from TOM-based connectivities after bootstrap inference (the more connectivity, the smaller the rank). Red: hub genes (ranked in top 5%, cf. S3 Fig).
(XLSX)

S4 Table A full list of DE genes between control and Sox6 KO mice hearts. (XLSX)

S5 Table Merged expression data of this study and O’Meara et al.’s study. Data from this study and O’Meara et al. (2015) (“1A_FPKM” sheet of Online Table I) are merged using gene symbol, and mean intensity values/FPKM values and standard deviations are shown.
(XLSX)
