# Supplementary figures and images for: Transcriptome Dynamics and Potential Roles of Sox6 in the Postnatal Heart
Source: PLoS One. 2016 Nov 10;11(11):e0166574. doi: 10.1371/journal.pone.0166574 (PMC5104335; doi:10.1371/journal.pone.0166574)

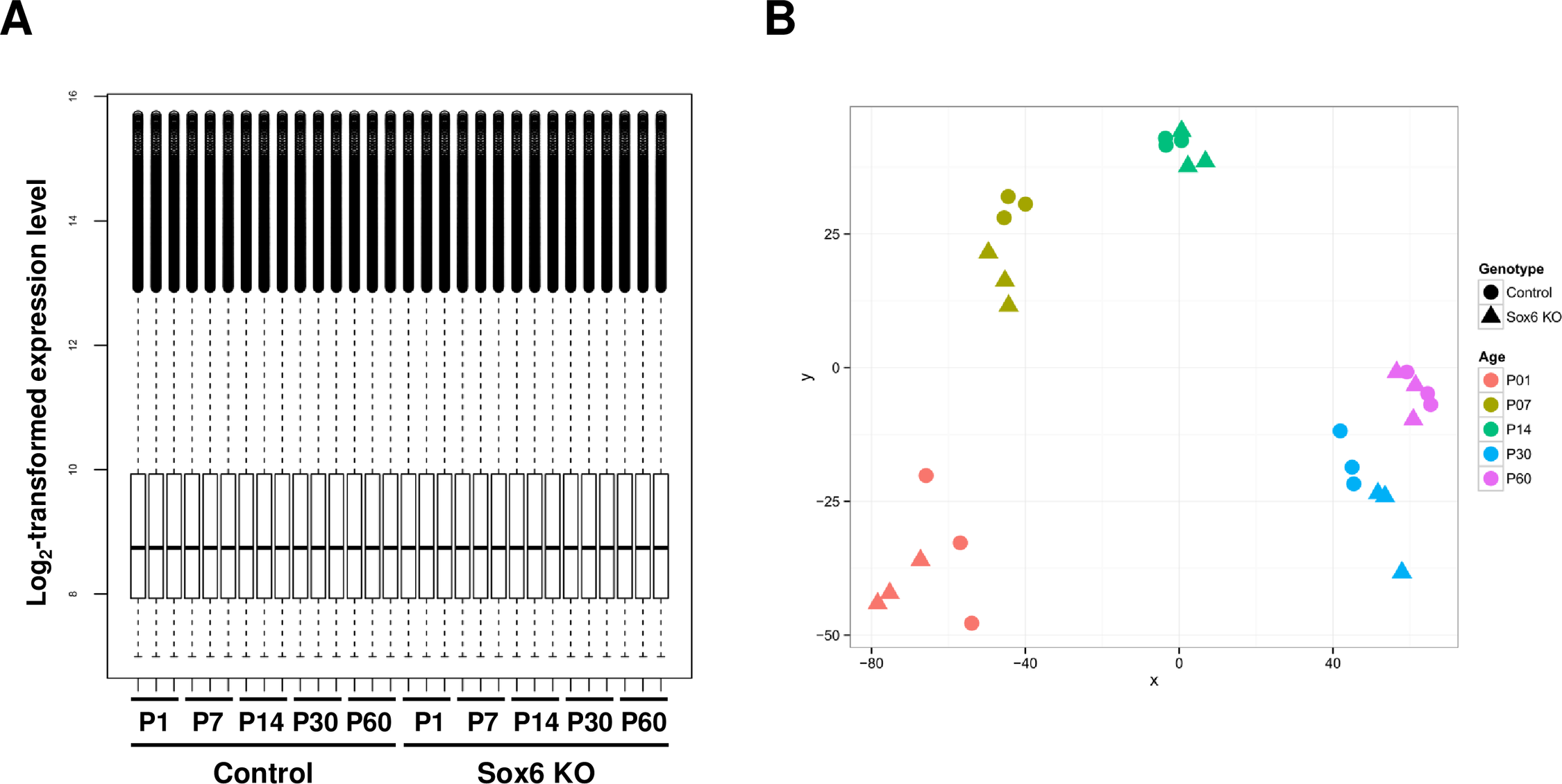

Supplement: S1 Fig — (A) A box plot showing normalized microarray data from triplicate samples at each developmental stage. (B) A multidimensional scaling (MDS) plot demonstrating similarity between each genotype and each developmental stage. Each axis represents an arbitrary unit and is therefore dimensionless. (TIF) [file pone.0166574.s001.tif]

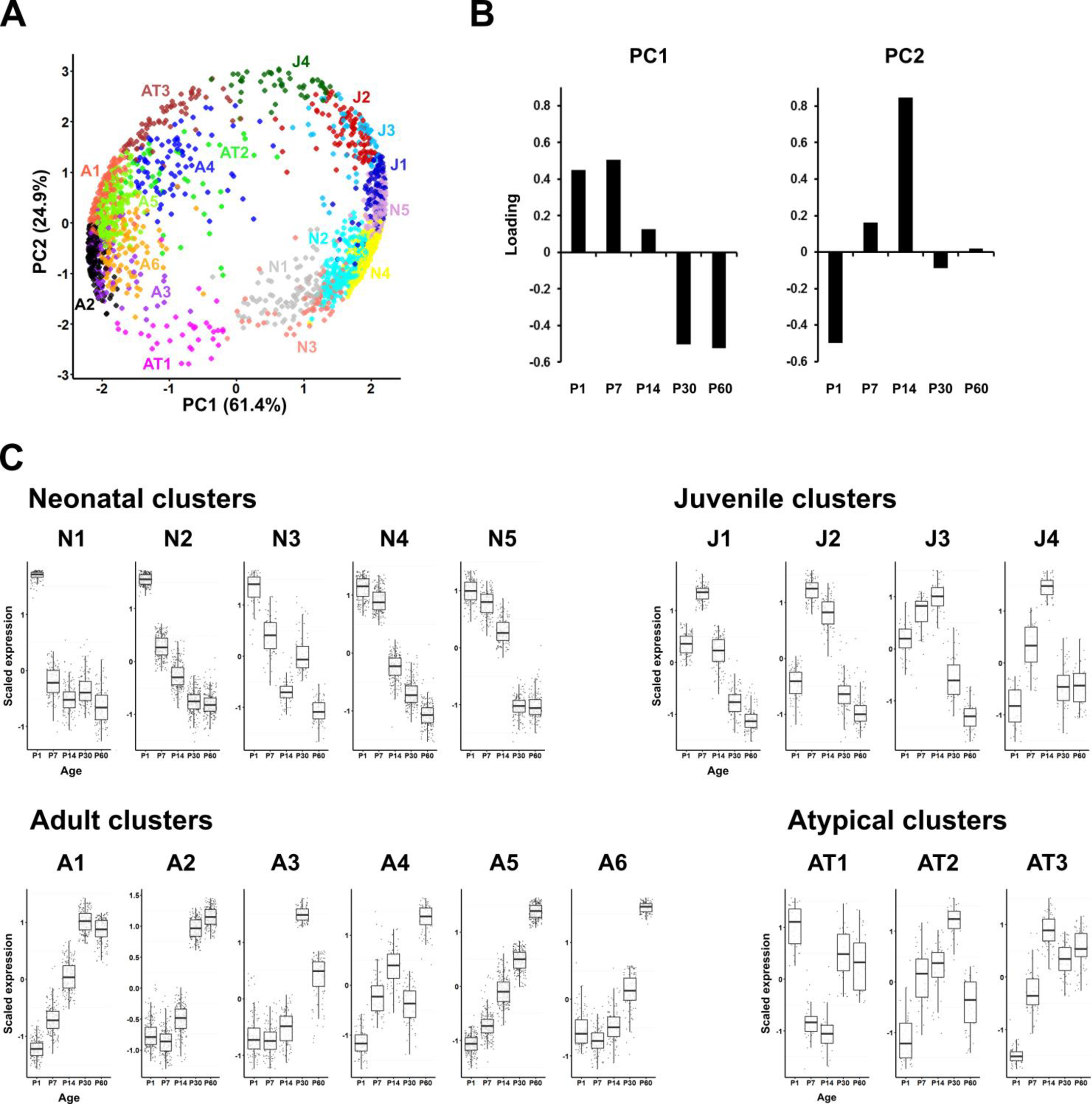

Supplement: S2 Fig — (A) PCA with SOM clustering of gene expression. The expression profile of each gene is represented, and genes that belong to different clusters are indicated by different colors and separated by PC1 and PC2. (B) Loading plots for PC1 and PC2 showing contributions of each variable (developmental stage) to the primary components. (C) Expression profiles of each SOM cluster. Scaled expression along developmental stages is shown in box plots together with cluster categories and names (Neonatal clusters: N1-N5; Juvenile clusters: J1-J4; Adult clusters, A1-A6; Atypical clusters: AT1-AT3). Of note, the SOM clusters obtained with mean intensity values were comparable but slightly different from those with median intensity values (Fig 1), suggesting that there were some variations/noises in intensity values of three biological replicates and they slightly affected clustering of genes. (TIF) [file pone.0166574.s002.tif]

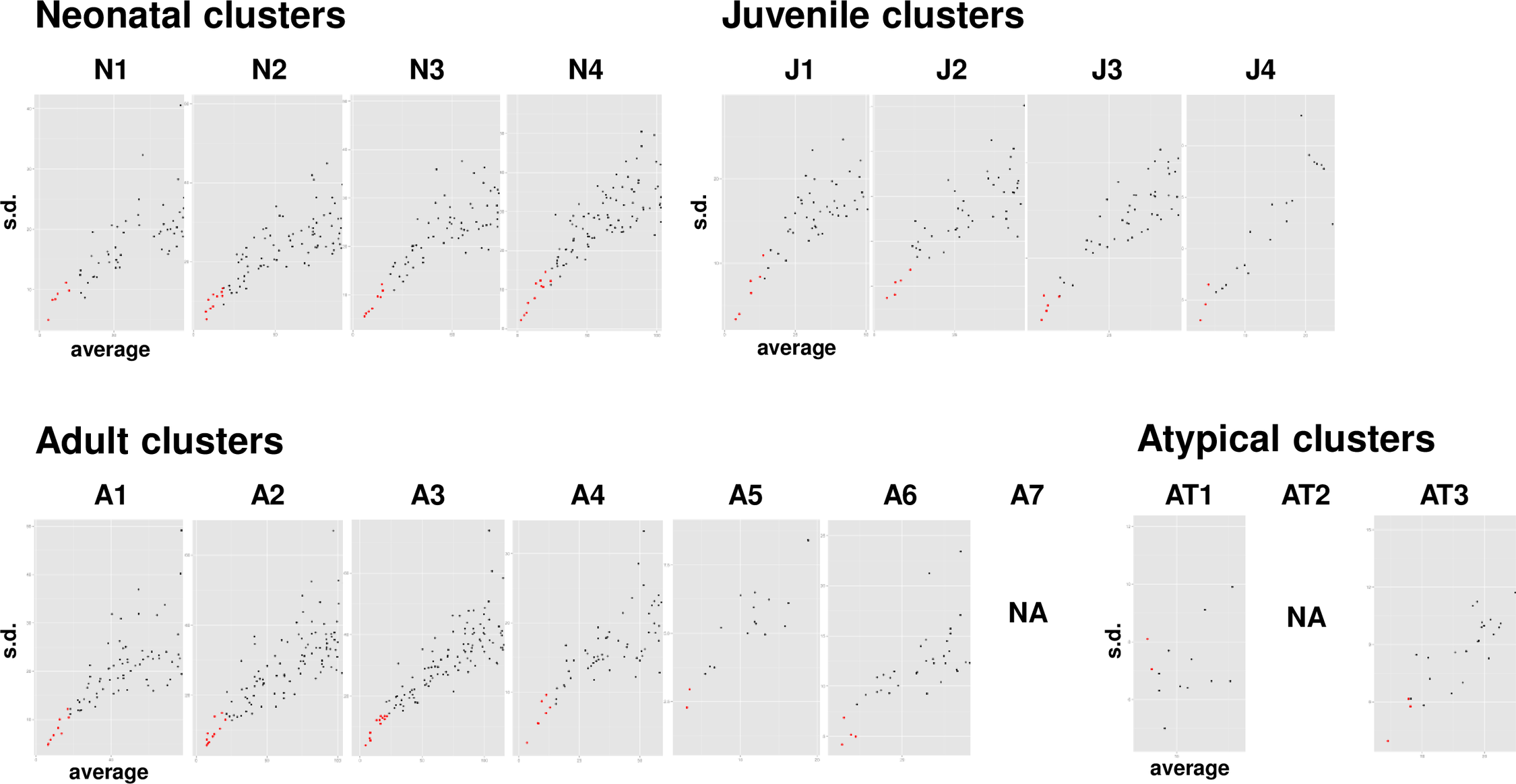

Supplement: S3 Fig — Genes were sorted according to the average ranking calculated from TOM-based connectivities after bootstrap inference (the more connectivity, the smaller the rank), and plotted against standard deviation (s.d.) of the ranks. Each dot represents individual gene. Genes ranked in top 50% in each SOM cluster are shown. Red dots: hub genes (ranked in top 5%). Black dots: other genes. (TIF) [file pone.0166574.s003.tif]

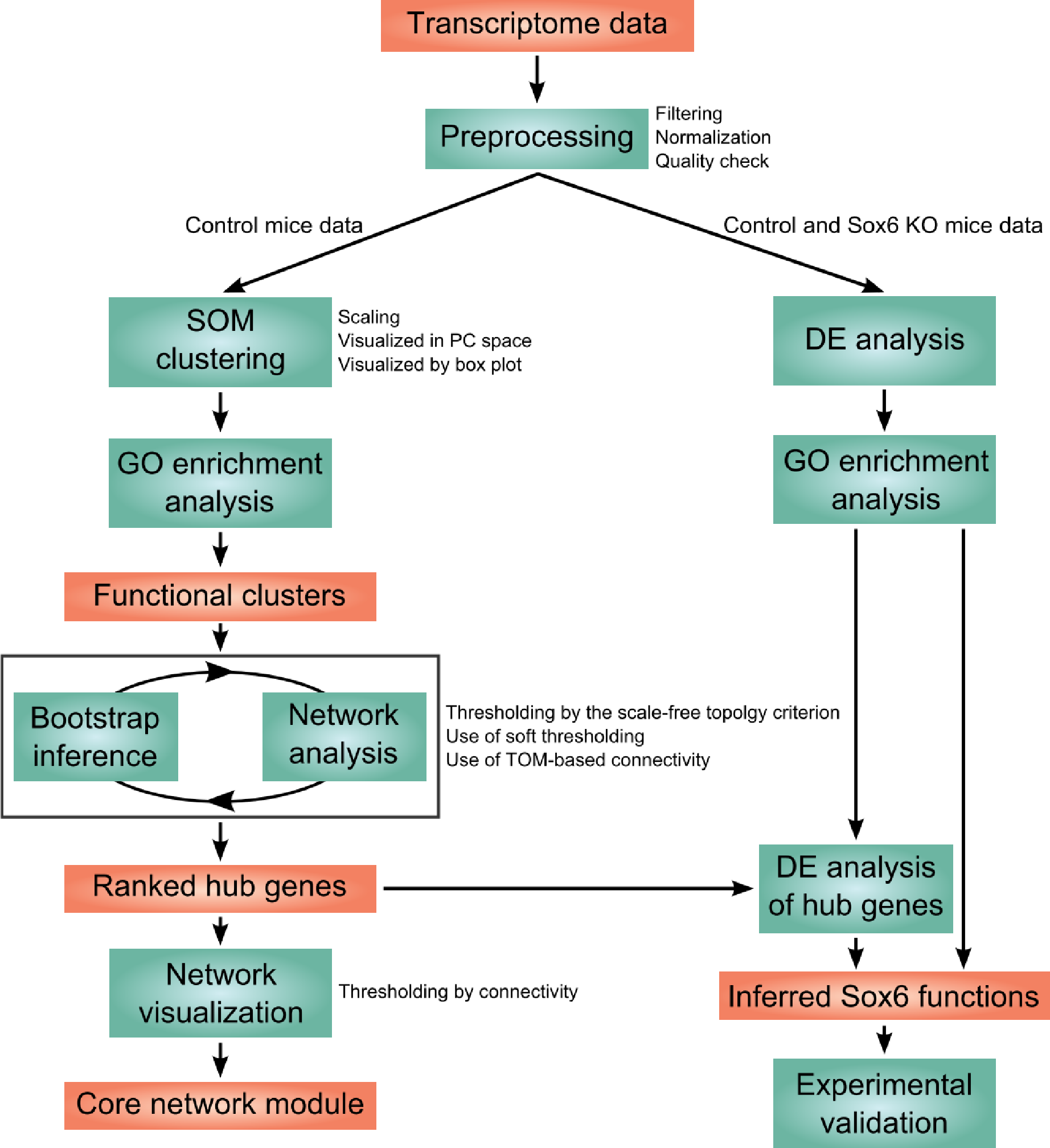

Supplement: S4 Fig — Workflow for analyzing control and Sox6 KO mice is shown. Data and results are shown in red, and processing steps are shown in green. (TIF) [file pone.0166574.s004.tif]

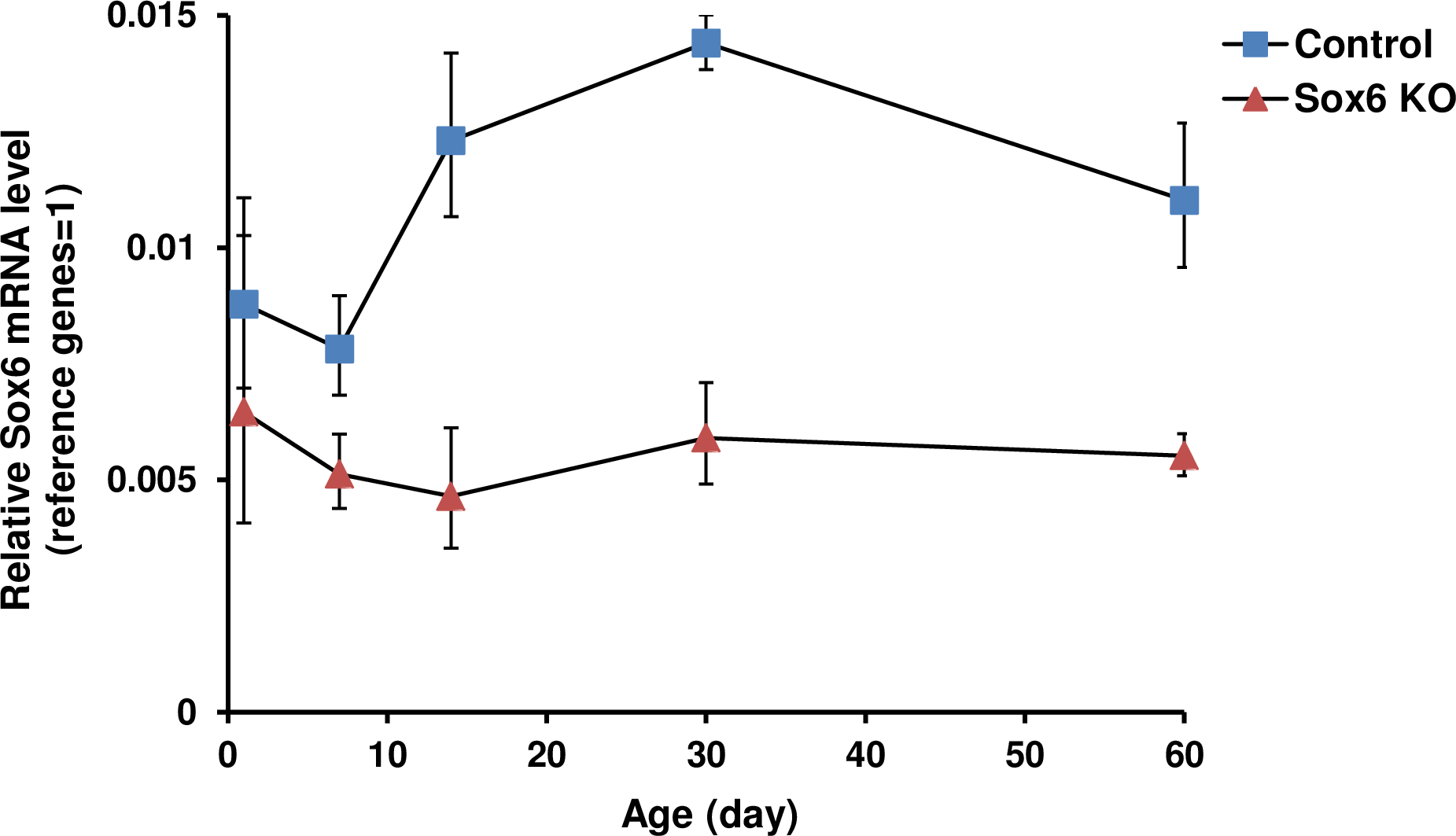

Supplement: S5 Fig — Total RNA was extracted from control and Sox6 KO mice ventricles, and Sox6 mRNA levels were quantified by RT-qPCR. Data are normalized for the reference genes (see the Methods section for details) and represented as mean ± s.d. (n = 3). (TIF) [file pone.0166574.s005.tif]

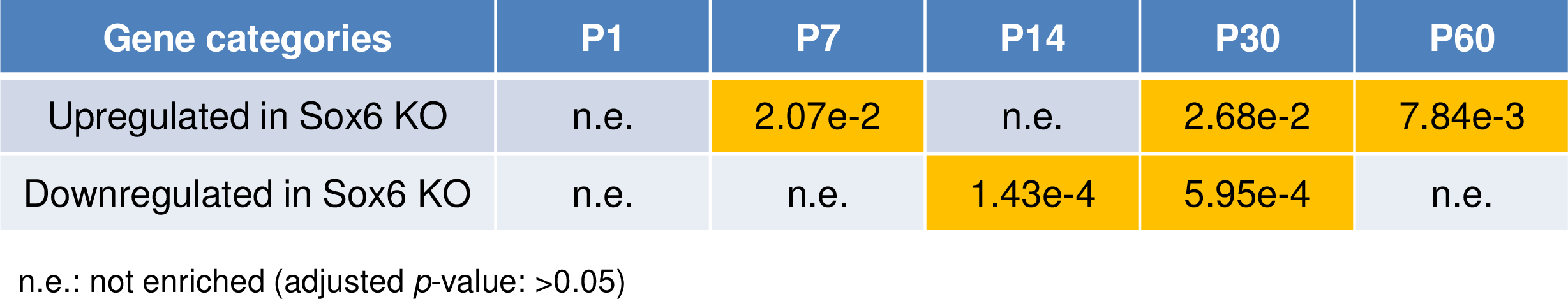

Supplement: S6 Fig — Significant enrichment of the Sox6 binding motif is shown as adjusted p-values (highlighted) obtained by AME. (TIF) [file pone.0166574.s006.tif]

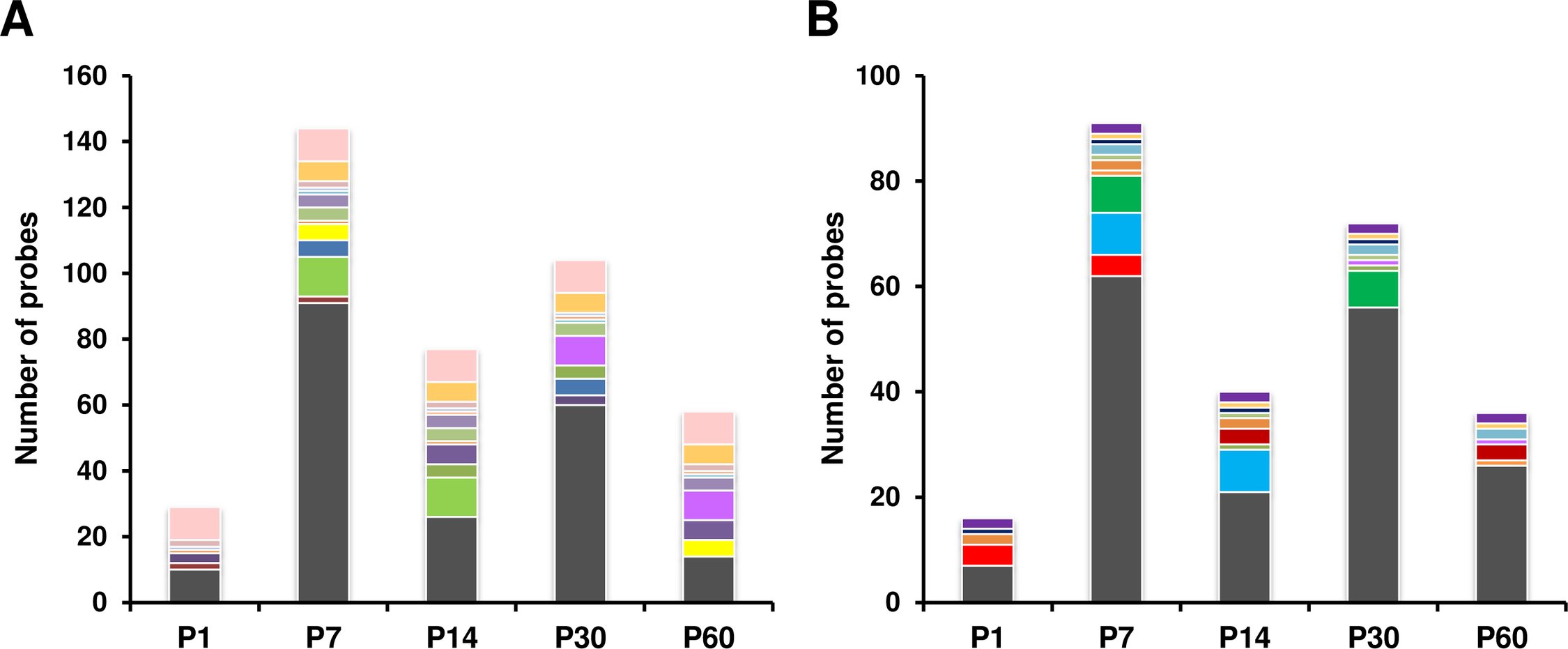

Supplement: S7 Fig — The number of probes for DE genes was counted at each time point and is shown as bar graphs. DE genes unique to each time point are shown in dark gray, and DE genes at multiple time points are shown in color (i.e. the same color indicates the same set of genes). (A) Upregulated genes in the Sox6 KO heart. (B) Downregulated genes in the Sox6 KO heart. (TIF) [file pone.0166574.s007.tif]

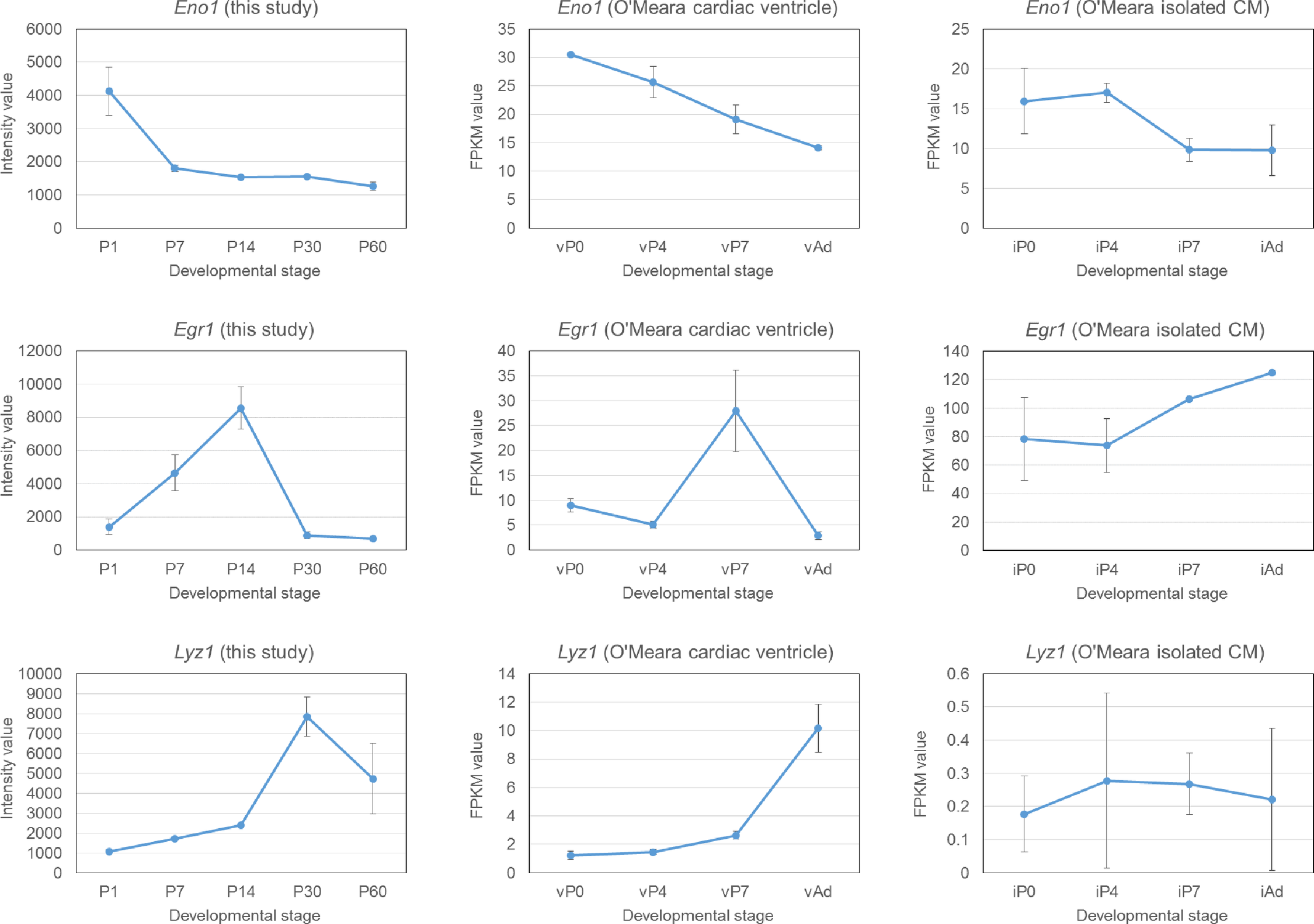

Supplement: S8 Fig — Expression profiles of three hub genes from different SOM clusters (Eno1 from cluster N1, Egr1 from cluster J4, and Lyz1 from cluster A4) obtained by this study and O’Meara et al. (2015) are exemplified (graphs are generated using S5 Table). As for the data from O’Meara et al. (2015), both tissue (cardiac ventricle)-derived data (vP0, vP4, vP7, and vAd data in S5 Table) and isolated cardiomyocyte (CM)-derived data (iP0, iP4, iP7, and iAd data in S5 Table) are shown. Eno1 showed similar expression patterns between iCM and cardiac ventricle, suggesting that Eno1 expression is similar between CM and other types of cells in cardiac ventricle. In contrast, Egr1 and Lyz1 showed distinct expression patterns between iCM and cardiac ventricle, suggesting that expression of Egr1 and Lyz1 is different between CM and other types of cells in cardiac ventricle. (TIF) [file pone.0166574.s008.tif]
